# Knowledge and practices related to non-alcoholic fatty liver disease among university students in Bangladesh

**DOI:** 10.1016/j.dialog.2026.100334

**Published:** 2026-08-04

**Authors:** Salma Khatun, Iffat Tasnim Haque

**Affiliations:** a American International University-Bangladesh, Dhaka, Bangladesh; b Islamic University of Technology, Gazipur, Bangladesh

**Keywords:** Non-alcoholic fatty liver disease, Knowledge and practice, University students, Lifestyle risk factors, Bangladesh

## Abstract

Non-alcoholic fatty liver disease (NAFLD) is a growing public health concern associated with obesity, diabetes, and metabolic syndromes. However, awareness and preventive behaviors remain understudied among young adults in low- and middle-income countries. This cross-sectional study assesses knowledge, attitudes, and practices related to NAFLD. This study is carried out among 343 university students (predominantly undergraduate students, mean age 24.6 years) at the Islamic University of Technology(IUT), Bangladesh. Even with 76.1% awareness, knowledge was very poor (39.1%) or moderate (40.2%). This shows significant gaps in understanding risk factors, treatment, prevention, and a strong dependence on informal information sources. Knowledge was particularly associated with age, region, and educational level. Preventive measures were extremely low, with only 11.1% followed by dieting restrictions and 31.2% making lifestyle changes. In an exploratory analysis limited to the 59 students who *self-reported* a prior NAFLD diagnosis (a figure likely inflated by over-reporting and not clinically confirmed), nearly half (47.5%) showed poor management practices. These findings reveal significant gaps in NAFLD literacy and health behaviors among university students in Bangladesh. This underscores the urgent need for institution-based and evidence-based educational programs and awareness campaigns to promote early prevention and effective self management of NAFLD.

## 1 Introduction

Non-alcoholic fatty liver disease (NAFLD) has emerged as a significant global health issue, representing the most common cause of chronic liver disease worldwide [1]. Its prevalence is increasing in parallel with obesity and type 2 diabetes mellitus, affecting an estimated 25% of the global population [2]. In Bangladesh, NAFLD is increasingly recognized as a major public health challenge. Studies indicate a substantial prevalence, with estimates varying but often exceeding 30% in certain populations, highlighting its significance even in non-Western settings [3]. The spectrum of NAFLD ranges from simple steatosis (fat accumulation in the liver) to non-alcoholic steatohepatitis (NASH), which involves inflammation and hepatocyte injury, which can progress to fibrosis, cirrhosis, and hepatocellular carcinoma. Beyond liver-related morbidity and mortality, NAFLD is strongly associated with cardiovascular disease, type 2 diabetes, and metabolic syndrome, contributing significantly to the overall health burden [4], [5].

Despite its high prevalence and potential severity, public awareness and understanding of NAFLD remain generally low worldwide [6], [7], and specific data for younger populations such as university students, especially in countries like Bangladesh, are limited [8], [9], [10]. This lack of awareness is concerning, as knowledge is considered a prerequisite for adopting preventive health behaviors [6]. University students, who are predominantly young adults (here defined as individuals aged approximately 18–35 years), represent a critical demographic for health interventions because they are establishing lifelong health habits and may serve as future health advocates or professionals [8], [11]. Understanding their current knowledge, attitudes, and practices regarding NAFLD is essential for developing effective prevention and management strategies tailored to this group. Previous research in Bangladesh has focused primarily on prevalence and risk factors in the general population or specific clinical groups [3], [4], with limited data on awareness within the university student community.

This study sought to fill this gap by evaluating students’ knowledge, attitudes, and practices regarding NAFLD at the Islamic University of Technology (IUT), a technically oriented, residential university in Bangladesh with a demographically diverse (national and international) student body. The objectives were to quantify the level of awareness regarding NAFLD’s causes, risk factors, consequences, prevention, and management; to explore the sources of information students rely on; to identify associations between socio-demographic factors and knowledge levels; and to understand current practices related to NAFLD prevention and management among this population. By providing insights into the current state of NAFLD awareness at IUT, this research seeks to inform the development of targeted educational programs and health-promotion initiatives aimed at mitigating the impact of NAFLD within the university community and potentially serving as a model for other institutions.

## 2 Methods

### 2.1 Study design

**Fig. 1:**
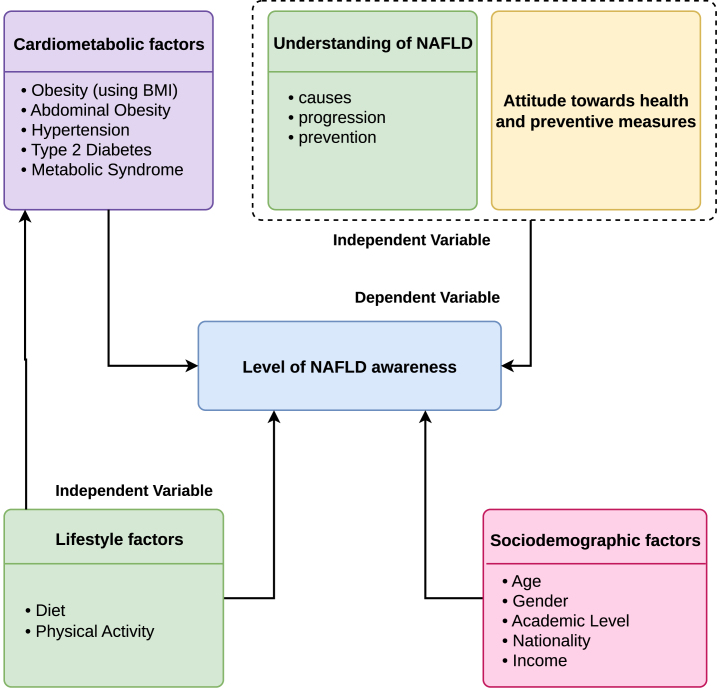
Conceptual framework illustrating the relationships between sociodemographic factors, lifestyle factors, cardiometabolic factors, understanding of NAFLD, attitudes, and the level of NAFLD awareness.

A descriptive cross-sectional study was conducted using a self-administered structured questionnaire to assess socio-demographic characteristics and NAFLD-related knowledge, attitudes, and practices among students at the Islamic University of Technology (IUT). The study followed a conceptual framework illustrating relationships between sociodemographic, lifestyle, cardiometabolic factors, and NAFLD awareness (Fig. 1).

### 2.2 Study population and site

The study population comprised undergraduate, Master’s, and Ph.D. students, both national and international, enrolled at IUT, located in Gazipur, Bangladesh. Staff and faculty were excluded. Nationality was self-reported as a demographic item; for analysis, the authors grouped the reported countries of origin into broader regions (Bangladeshi, African, Asian, Middle Eastern, and North American), so that “region” is an author-derived grouping rather than a separate questionnaire item.

### 2.3 Eligibility criteria

Inclusion criteria encompassed all currently enrolled male and female undergraduate, Master’s, and Ph.D. students at IUT. Exclusion criteria included students who did not provide consent, as well as university staff and faculty members.

### 2.4 Sample size calculation

As no prior studies specifically assessed NAFLD awareness among private university students in this context, Cochran’s formula was used for sample size calculation. Based on a previously reported fatty liver prevalence of 34.34% in a nationwide Bangladeshi study [12], a 95% confidence level (Z = 1.96), and a 5% margin of error (d = 0.05), the required sample size was calculated as n = [Z${}^{2}$ * P * (1-P)]/d${}^{2}$ = [(1.96)${}^{2}$ * 0.3434 * (1 − 0.3434)]/(0.05)${}^{2}$ 347. Adjusting for an estimated 10% non-response rate, the target sample size was 381.

### 2.5 Study sample and period

Due to time and resource constraints during the study period, a final sample of 343 completed surveys was obtained and included in the analysis. Data collection occurred between September 2024 and July 2025.

### 2.6 Research instrument

A structured questionnaire was developed based on literature review and expert consultation. It included sections on socio- demographic information (age, gender, nationality, educational qualification), knowledge about NAFLD (12 questions covering awareness, causes, progression, treatment, prevention, diagnosis), and attitudes/practices (6 questions including diagnosis history, dietary restrictions, comorbidities, medication use, lifestyle changes, perceived improvement). The questionnaire was pre-tested on a small sample of students to ensure clarity and relevance, and revisions were made based on feedback. Content validity was established through review by two public health experts and one hepatologist. Face validity was confirmed during pre-testing with 15 students (not included in the final sample). Internal consistency was not calculated as most items were single, categorical knowledge questions rather than a psychometric scale.

### 2.7 Data collection

Quantitative data were collected using a pre-tested, self- administered questionnaire in English, which is the medium of instruction at IUT and the common language among its national and international students. Eligible students were approached on campus and through university communication channels and invited to participate; participation was entirely voluntary. The questionnaire was distributed as a paper-based self-administered survey, and written informed consent was obtained from every participant before survey administration. Of the 384 students invited, 343 returned complete responses, yielding a response rate of 89.3%. A copy of the questionnaire and the informed-consent form is provided as Supplementary Material.

### 2.8 Data analysis

Data were entered and analyzed using IBM SPSS Statistics. Descriptive statistics (frequencies, percentages, mean, standard deviation) were used to summarize socio-demographic characteristics and responses to knowledge, attitude, and practice questions. The knowledge score was derived from the 12 knowledge questions; the question on the source of information was not scored. One point was awarded for each affirmative or correct response to nine single-answer items: having heard of fatty liver, having heard of cirrhosis, believing fatty liver to be hereditary, treatment availability, preventability, occurrence in non-alcoholics, early-stage curability, correct identification of liver sonogram as the diagnostic method, and recognition that fatty liver can cause serious health problems. For the two multiple-selection questions (conditions that can cause fatty liver, and conditions that can advance to cirrhosis), 0.25 points were awarded for each correct option, up to a maximum of 1 point per question. The maximum attainable score was therefore 11. Participants were classified a priori, using thresholds of 50% and 75% of this maximum, as having Poor (score < 5.5, i.e. below 50%), Moderate (5.5 to <8.25, 50%–75%), or Strong (score ≥ 8.25, ≥75%) knowledge.

Among the 59 participants who self-reported a NAFLD diagnosis, practice level was dichotomized as “Good” or “Poor” based on three binary items: (1) following specific dietary restrictions for NAFLD, (2) making lifestyle changes to manage/reduce risk of NAFLD, and (3) reporting improvement after lifestyle changes (if applicable). Participants who answered “Yes” to at least two of these items were classified as having Good Practice; others were classified as Poor Practice.

Cross-tabulations and Pearson’s Chi-square tests were performed to assess associations between socio-demographic variables (age group, gender, region, educational qualification) and knowledge levels, as well as between these variables and practice levels among diagnosed patients. A p-value < 0.05 was considered statistically significant. Missing responses were handled by available-case (pairwise) analysis; the valid denominator is reported for each item, and no imputation was performed.

### 2.9 Data quality management

Measures included pre-testing the questionnaire, careful data entry with double-checking, prompt handling of inconsistencies, ensuring participant anonymity and confidentiality, secure data storage, and regular monitoring of the data collection process to maintain methodological rigor.

### 2.10 Ethical considerations

The study strictly followed ethical protocols, and informed consent was secured from all participants, ensuring voluntariness, anonymity, and the right to withdraw. Confidentiality was maintained throughout the study. Verbal approval was obtained from the Ethical Review Board (ERB) of the Islamic University of Technology (IUT) prior to commencing data collection. The Board subsequently granted formal ethical approval for the study on a *retrospective* basis and issued an approval reference (EA_21_07_2026_01, dated 21 July 2026). As the Board records approvals by reference number rather than issuing a separate approval letter, this approval can be independently verified by contacting the ERB directly (head.reasp@iut-dhaka.edu). We explicitly acknowledge that prospective written ethical approval was not obtained before data collection began, and this is stated as a limitation of the study (Section 5). Written informed consent was obtained from every participant before survey administration.

## 3 Results

A university-based descriptive analysis was conducted to assess the knowledge and awareness of Non-Alcoholic Fatty Liver Disease (NAFLD) among students at the Islamic University of Technology. A total of 343 respondents participated via a self-administered structured questionnaire. The analysis began with an examination of missing values. Significant missing data were noted for questions regarding the source of information about fatty liver (82 missing, 23.9%), conditions believed to advance to cirrhosis (65 missing, 19.0%), and adherence to specific dietary restrictions for NAFLD (32 missing, 9.3%). These missing values were considered in subsequent analyses.

**Table 1:** Association between Socio-demographic Factors and NAFLD Knowledge Level (n = 343).

Variable	Category	Poor knowledge	Moderate knowledge	Strong knowledge	Total	Chi-Square (X${}^{2}$)	P-value
Age group (Years)	<20	33 (46.5%)	25 (35.2%)	13 (18.3%)	71 (100%)	14.75 (df = 6)	0.022
	20–30	90 (36.9%)	108 (44.3%)	46 (18.9%)	244 (100%)		
	31–40	8 (42.1%)	4 (21.1%)	7 (36.8%)	19 (100%)		
	41–50	3 (33.3%)	1 (11.1%)	5 (55.6%)	9 (100%)		

Gender	Male	71 (34.6%)	91 (44.4%)	43 (21.0%)	205 (100%)	4.77 (df = 2)	0.092
	Female	63 (45.7%)	47 (34.1%)	28 (20.3%)	138 (100%)		

Nationality/Region	Bangladeshi	97 (35.9%)	120 (44.4%)	53 (19.6%)	270 (100%)	21.69 (df = 8)	0.006
	African	23 (47.9%)	14 (29.2%)	11 (22.9%)	48 (100%)		
	Asian	7 (70.0%)	2 (20.0%)	1 (10.0%)	10 (100%)		
	Middle Eastern	6 (60.0%)	2 (20.0%)	2 (20.0%)	10 (100%)		
	North American	1 (20.0%)	0 (0.0%)	4 (80.0%)	5 (100%)		

Educational qualification	Undergraduate	113 (38.8%)	126 (43.3%)	52 (17.9%)	291 (100%)	16.10 (df = 4)	0.003
	Master’s	18 (42.9%)	11 (26.2%)	13 (31.0%)	42 (100%)		
	PhD	3 (30.0%)	1 (10.0%)	6 (60.0%)	10 (100%)

**Table 2:** Perceived conditions leading to cirrhosis (Multiple responses allowed, n = 278 valid responses).

Perceived condition(s)	Frequency	% of Valid responses
Alcohol consumption only	75	27.0
Fatty liver only	69	24.8
Hepatitis B infection only	31	11.2
Hepatitis C infection only	22	7.9
Alcohol consumption + Fatty liver	26	9.4
Alcohol consumption + Hepatitis C infection	16	5.8
Alcohol consumption + Fatty liver + Hepatitis C	9	3.2
Combinations involving alcohol/Fatty liver/Hep	30	10.8

### 3.1 Socio-demographic characteristics

The socio-demographic analysis revealed a diverse participant pool, as detailed in Table 4. The majority of respondents were Bangladeshi (270, 78.7%), followed by African (48, 14.0%), Asian (10, 2.9%), Middle Eastern (10, 2.9%), and North American (5, 1.5%). Educational qualifications were predominantly undergraduate (291, 84.8%), with smaller proportions holding Master’s (42, 12.2%) or PhD degrees (10, 3.0%). The age distribution was skewed towards younger adults, with the largest group aged 20–30 years (244, 71.1%), followed by those under 20 years (71, 20.7%), 31–40 years (19, 5.5%), and 41–50 years (9, 2.6%). The mean age was 24.63 years (SD = 5.11). Gender distribution showed a higher participation rate among males (205, 59.8%) compared to females (138, 40.2%).^1^

Knowledge Regarding NAFLD: Analysis of knowledge relevant to NAFLD indicated that a majority (261, 76.1%) had heard about fatty liver, while 82 (23.9%) had not. Among those aware, the primary sources of information (Table 3) were relatives (102, 39.1%), social media (73, 28.0%), doctors (46, 17.6%), and Google (40, 15.3%).

Familiarity with cirrhosis was reported by 59.2% (203) of participants. When asked about conditions leading to cirrhosis (Table 2), alcohol consumption (75, 27.0% of valid responses) and fatty liver (69, 24.8%) were the most commonly identified single factors. Combinations involving these and hepatitis infections were mentioned less frequently.^2^,^3^

**Table 3:** Sources of information about fatty liver among aware participants (n = 261).

Source	Frequency	Percent
Relatives	102	39.1
Social Media	73	28.0
Doctor	46	17.6
Google	40	15.3

Regarding conditions potentially causing fatty liver, high cholesterol (identified in combinations totaling 44.6% of responses) and excess alcohol intake (44.0%) were frequently cited, followed by obesity (38.2%) and diabetes (23.0%).

Participants’ perceptions on various aspects of NAFLD were explored. A belief in the hereditary nature of fatty liver was held by 51.0% (175), while 28.0% (96) disagreed and 21.0% (72) were unsure. Regarding treatment availability, responses were mixed: 28.3% (97) believed treatment exists, 29.4% (101) believed it does not, and 42.3% (145) were uncertain. A majority (53.9%, 185) believed NAFLD is preventable. Awareness that NAFLD can occur in non-alcoholics was high (68.8%, 236). Belief in the possibility of curing NAFLD in its early stages was held by 58.3% (200). A significant majority (69.7%, 239) perceived NAFLD as capable of causing serious health problems. For diagnosis, liver sonogram was the most recognized method (189, 55.1%), followed by body weight/obesity (100, 29.2%) and blood tests (36, 10.5%).

**Table 4:** Socio-demographic characteristics of study participants (n = 343).

Characteristic	Category	Frequency	Percent
Nationality	Bangladeshi	270	78.7
	African	48	14.0
	Asian	10	2.9
	Middle Eastern	10	2.9
	North American	5	1.5

Educational qualification	Undergraduate	291	84.8
	Master’s	42	12.2
	PhD	10	3.0

Age group (Years)	<20	71	20.7
	20–30	244	71.1
	31–40	19	5.5
	41–50	9	2.6

	Mean Age (SD)	24.63	(5.11)

Gender	Male	205	59.8
	Female	138	40.2

### 3.2 Attitudes and practices

Analysis of attitudes and practices revealed that 17.2% (59) of participants reported having been diagnosed with NAFLD, while 77.6% (266) had not, and 5.2% (18) were unsure. Regarding practices, only 11.1% (38) reported following specific dietary restrictions for NAFLD, while 70.6% (242) did not. Existing related medical conditions (like diabetes or hypertension) were reported by 21.3% (73). Similarly, 21.3% (73) reported taking medications or supplements for liver health or NAFLD management. Lifestyle changes to reduce risk or manage NAFLD were reported by 31.2% (107). Among those who made changes, 26.8% (92 of the total sample, or 86% of those who made changes) noticed improvements.

### 3.3 NAFLD knowledge score and associations

A knowledge score was calculated based on the 12 knowledge questions. The distribution showed 39.1% (134) with Poor Knowledge (score < 5.5), 40.2% (138) with Moderate Knowledge (score 5.5 to <8.25), and 20.7% (71) with Strong Knowledge (score ≥ 8.25). Chi-square tests (Table 1) revealed significant associations between knowledge level and age group (p = 0.022), region (p = 0.006), and educational qualification (p = 0.003). Older age groups (41–50 years showing 55.6% strong knowledge) and higher educational qualifications (PhD holders showing 60% strong knowledge) were associated with better knowledge. Regional differences were notable, with North Americans showing the highest proportion of strong knowledge (80%), while Asians showed the highest proportion of poor knowledge (70%). Gender was not significantly associated with knowledge level (p = 0.092).

**Table 5:** Practices among participants self-reporting a NAFLD diagnosis (n = 59; exploratory).

Variables	Category	Poor practice	Good practice	Total	Chi-Square (X${}^{2}$)	P-value
Age group (Years)	<20	4 (33.3%)	8 (66.7%)	12 (100%)	1.69 (df = 3)	0.640
	20–30	19 (51.4%)	18 (48.6%)	37 (100%)		
	31–40	3 (42.9%)	4 (57.1%)	7 (100%)		
	41–50	2 (66.7%)	1 (33.3%)	3 (100%)		
	Total	28 (47.5%)	31 (52.5%)	59 (100%)		

Gender	Male	19 (51.4%)	18 (48.6%)	37 (100%)	0.60 (df = 1)	0.437
	Female	9 (40.9%)	13 (59.1%)	22 (100%)		
	Total	28 (47.5%)	31 (52.5%)	59 (100%)		

Region	African	3 (42.9%)	4 (57.1%)	7 (100%)	0.53 (df = 4)	0.971
	Asian	1 (50.0%)	1 (50.0%)	2 (100%)		
	Bangladeshi	21 (50.0%)	21 (50.0%)	42 (100%)		
	Middle Eastern	2 (40.0%)	3 (60.0%)	5 (100%)		
	North American	1 (33.3%)	2 (66.7%)	3 (100%)		
	Total	28 (47.5%)	31 (52.5%)	59 (100%)		

Educational qualification	Undergraduate	22 (55.0%)	18 (45.0%)	40 (100%)	3.52 (df = 2)	0.172
	Master’s	4 (26.7%)	11 (73.3%)	15 (100%)		
	PhD	2 (50.0%)	2 (50.0%)	4 (100%)		
	Total	28 (47.5%)	31 (52.5%)	59 (100%)		

### 3.4 Practices among diagnosed patients

Finally, an exploratory analysis of practices was conducted among the 59 participants who *self-reported* a prior NAFLD diagnosis (Table 5). Because these diagnoses were self-reported and not clinically confirmed by imaging, biomarkers, or medical records (see Section 5), all results for this subgroup should be interpreted as exploratory and hypothesis-generating rather than as reflecting clinically verified NAFLD. Practice level was dichotomized as ‘Good’ or ‘Poor’ based on three self-reported items (dietary restriction, lifestyle change, and perceived improvement), with participants answering ‘Yes’ to at least two classified as Good Practice. Overall, 47.5% (28) exhibited poor practice and 52.5% (31) good practice. None of the socio-demographic factors examined were significantly associated with practice level: age group (p = 0.640), gender (p = 0.437), region (p = 0.971), and educational qualification (p = 0.172). Descriptively, Master’s-level students showed the highest proportion of good practice (73.3%) compared with undergraduates (45.0%) and Ph.D. students (50.0%), but, given the small number of diagnosed participants in each subgroup, this difference did not reach statistical significance and should be interpreted with caution.^4^

## 4 Discussion

### 4.1 Principal findings

This study assessed the awareness, knowledge, and understanding of Non-Alcoholic Fatty Liver Disease (NAFLD) among students at the Islamic University of Technology (IUT). The findings indicate a foundational level of awareness, with approximately 76% of participants having heard of fatty liver. However, this awareness primarily stems from informal channels like relatives (39.1%) and social media (28.0%), rather than authoritative sources like doctors (17.6%), as shown in Table 3.

### 4.2 Awareness, information sources, and knowledge gaps

This reliance on informal sources may contribute to the observed gaps in detailed knowledge and potential misconceptions. This finding is consistent with studies in other regions; for example, Du et al. (2023) found low NAFLD awareness (26.2%) among Chinese university students [8], and Lee et al. (2023) reported unsatisfactory awareness in the South Korean general population [7]. A recent study among Lebanese university students similarly reported that more than half (54.7%) had no prior knowledge of metabolic dysfunction-associated fatty liver disease [10].

While basic awareness exists, deeper knowledge regarding NAFLD specifics, such as its progression to cirrhosis (Table 2), treatment availability, and diagnostic methods, reveals considerable uncertainty. For instance, a large proportion (42.3%) were unsure about treatment availability, and while 55.1% correctly identified sonography for diagnosis, a considerable number (29.2%) incorrectly associated diagnosis primarily with body weight or obesity. This highlights a discrepancy between recognizing the term “fatty liver” and understanding its clinical implications, a finding echoed in other health-literacy studies among young adults [6].

### 4.3 Socio-demographic correlates of knowledge

Knowledge level was significantly associated with several socio-demographic factors (Table 1). Individuals with higher levels of education (Master’s and PhD) and older age groups (31–50 years) showed stronger knowledge, suggesting that advanced education and increased life experience may contribute to better health literacy regarding NAFLD. This aligns with findings by Du et al. (2023), where medical background and higher medical knowledge were associated with better NAFLD awareness in Chinese students [8].

The strong knowledge observed in North American students (80%), although based on a very small subgroup (n = 5), warrants cautious interpretation and further investigation into potential cultural or educational-system differences in health-information dissemination. Conversely, the high proportion of poor knowledge among Asian students (70%, n = 10) may suggest a need for targeted educational interventions, although this too is based on a small subgroup and should be interpreted with caution. The lack of a significant gender difference (p = 0.092) contrasts with some studies but aligns with others, including Noor et al. (2023), who found no significant gender difference in NAFLD knowledge among Malaysian pharmacy and medical students [9], indicating that gender’s role in health knowledge may be context-specific.

### 4.4 Preventive practices among diagnosed and at-risk students

A notable finding regarding practices was the low adoption of preventive measures, even among those aware of the disease: only 11.1% of the total sample followed dietary restrictions related to NAFLD, and only 31.2% reported making lifestyle changes. This gap between awareness and action is a common challenge in health-behavior change [6].

Among the 59 students who *self-reported* a NAFLD diagnosis, nearly half (47.5%) showed poor management practices; however, because this diagnosis was self-reported and the 17.2% rate is biologically implausible for this young population (see Section 5), these subgroup findings are exploratory and must be interpreted with caution. No socio-demographic factor was significantly associated with practice level in this subgroup. The limited adoption of recommended practices despite a reported diagnosis may suggest barriers beyond knowledge, possibly including motivation, perceived severity, or access to resources and support, but this remains a hypothesis to be tested in studies using objectively confirmed diagnoses.

Educational qualification showed a descriptive gradient in self-management, with Master’s-level students reporting the highest proportion of good practice (73.3%) compared with undergraduates (45.0%) and Ph.D. students (50.0%); however, this association did not reach statistical significance (p = 0.172), and the small number of diagnosed participants in each subgroup limits interpretation. Likewise, practice level did not differ significantly by age group (p = 0.640), sex (p = 0.437), or region (p = 0.971). This contrasts with the knowledge findings, where region and education were significantly associated, and suggests that translating awareness into sustained preventive behavior may depend on factors not captured here, such as motivation, self-efficacy, and access to support, rather than socio-demographic characteristics alone. These results underscore the need for practice-focused interventions (e.g., goal-setting workshops, peer support groups, or behavioral coaching) for diagnosed and at-risk students, who appear to under-implement recommended changes despite varying levels of knowledge. The finding that 26.8% of the total sample (or 86% of those who made changes) noticed improvements after lifestyle modification is encouraging and demonstrates the potential efficacy of such interventions. However, the large proportion who did not perceive any improvement warrants attention; this may reflect insufficient or inappropriate changes, unrealistic expectations, the chronic nature of the condition, or the need for more intensive or medically supervised interventions.

### 4.5 The Bangladeshi context and broader implications

Comparing these findings with the background literature from Bangladesh reveals both contrasts and consistencies. The high prevalence of NAFLD risk factors such as obesity and diabetes reported in national studies [4], [12] underscores the importance of NAFLD awareness in this student population. However, the knowledge gaps observed here, particularly regarding non-alcoholic causes and preventability, suggest that national prevalence data may not translate directly into public understanding, especially among younger, educated populations who might underestimate their personal risk.

The high percentage (68.8%) that recognizes that NAFLD can occur in non-alcoholics is a positive result. But the significant proportion that relates it primarily with alcohol (44% identifying excess alcohol intake as a cause) shows persistent confusion that needs clarification through education. Educational efforts should highlight NAFLD as a distinct entity often linked to metabolic factors, not only alcohol. Because diet is a central modifiable driver of metabolic liver disease, such efforts should also address food choices and the potential role of fortified and functional foods in supporting healthier dietary patterns [13], [14].

Naturally, while students are aware of NAFLD, there are significant gaps in detailed knowledge, understanding of risk factors, prevention, and management. The dependence on informal information sources and the low adoption of preventive behaviors, even among diagnosed individuals, underscore the urgent need for structured evidence-based educational interventions and health promotion strategies within the university setting. Such interventions have shown promise in other groups of young adults [11].

## 5 Limitations

This research has several significant limitations that should be considered when interpreting the findings. First, the cross-sectional design limits the ability to establish temporal or causal relationships between knowledge levels, socio-demographic factors, and preventive practices. All data were self-reported, introducing potential recall bias, social desirability bias, and misinterpretation of questions, particularly because the questionnaire was administered in English in a multilingual university environment where many students’ first language may not be English.

Second, the self-reported rate of NAFLD 17.2% (59 out of 343 participants) is unusually high for a young university student population (mean age 24.63 years, predominantly 18–30 years). In most of the common young adult populations, NAFLD is largely asymptomatic and undiagnosed unless actively detected by imaging or biomarkers. This elevated rate likely reflects over-reporting, confusion with other liver-related findings (e.g., elevated enzymes), selection bias, or response error.

Third, the research was conducted in a single institution (the Islamic University of Technology, IUT) using convenience sampling due to time and resource constraints. This restricts the generalization of the findings to other universities, regions, or the wider population of Bangladeshi students. Moreover, the sample size was calculated from a reported fatty-liver prevalence rather than an expected awareness or knowledge level, and the achieved sample (n = 343) fell slightly below the calculated target (n = 347 before, and 381 after, adjustment for non-response); the study may therefore be modestly underpowered for some comparisons. Furthermore, the data was also unevenly distributed in key demographic variables: the vast majority were Bangladeshi (78.7%), undergraduate (84.8%) and aged 20–30 years (71.1%), with very small subgroups for Ph.D. students (n = 10), North American students (n = 5), and older age groups (41–50 years: n = 9). These small cell sizes reduced the statistical power of some subgroup analyses and may have contributed to unstable estimates in certain categories; accordingly, subgroup comparisons involving small groups (e.g., North American or Asian students, or diagnosed patients by educational level) should be regarded as exploratory.

Additionally, substantial missing data were observed for several questions (e.g., 23.9% missing for sources of information on fatty liver, 19.0% for conditions advancing to cirrhosis). Because each item was analyzed using the available (valid) responses, denominators varied across analyses; this available-case approach may have introduced bias or reduced precision for the affected items. The knowledge scoring system, although applied consistently, was developed for this study rather than adapted from a previously validated instrument; its cut-off points corresponded to 50% and 75% of the maximum score but were not benchmarked against an external standard. Similarly, the dichotomization of practice level among diagnosed participants was based on three self-reported items without external validation.

The study did not include objective measures of NAFLD (e.g., ultrasound, FibroScan, or liver function tests) or detailed dietary/physical activity assessments, limiting the ability to correlate perceived knowledge and practices with actual health status or behaviors. Relatedly, the self-reported NAFLD diagnosis was not clinically verified; consequently, all analyses of the diagnosed subgroup are exploratory and should not be interpreted as reflecting confirmed disease.

Finally, with respect to ethical oversight, data collection was initiated on the basis of verbal ERB approval, and formal written approval was obtained only retrospectively (Section 2.10). While the study adhered to the principles of voluntary participation, informed consent, anonymity, and confidentiality throughout, the absence of prospective written ethical approval is a limitation, and future studies should ensure that written approval is secured before data collection begins.

Despite these limitations, the study provides valuable preliminary information on NAFLD awareness and practices in a university setting in Bangladesh. This highlights important gaps that justifies further investigation in larger multi-center studies with mixed methods and objective verification.

## 6 Conclusion

This study identified substantial gaps in NAFLD knowledge among students at the Islamic University of Technology, despite a foundational level of awareness. Many students lacked a clear understanding of NAFLD’s metabolic risk factors, its potential for asymptomatic progression, and evidence-based prevention and management strategies, and most relied on informal information sources. Preventive practices were limited, even among students reporting a prior diagnosis. Comparable gaps have been reported in other young-adult populations [7], [8], [9], [10].

Given the rising prevalence of NAFLD [2] and its established links to serious health outcomes [4], [5], these gaps could plausibly contribute to delayed help-seeking and suboptimal self-management; however, the cross-sectional, self-reported design precludes causal inference, and such outcomes remain only suggestive. The findings support the need for structured, evidence-based educational initiatives (integrated into health curricula or delivered through workshops and credible digital channels, and developed in collaboration with healthcare professionals), alongside a supportive campus environment offering healthier food options, accessible fitness facilities, and counseling. By addressing these gaps, IUT and similar institutions can help students make informed health decisions and may help reduce the future burden of NAFLD in this population.

## 7 Recommendations

The findings of this research show a low depth of knowledge despite moderate awareness, a heavy dependence on informal sources, and a low adoption of preventive behaviors even among diagnosed students (47.5% poor practice). The unexpectedly high rate of self-reported NAFLD diagnosis (17.2%) further highlights the urgent need for targeted institution-specific interventions at IUT and similar technical universities in Bangladesh.

First, integrate structured NAFLD education into existing curriculum or orientation programs, non-alcoholic etiology, early-stage reversibility, and evidence-based lifestyle modifications. This would address the observed gaps in understanding the progression to cirrhosis and treatment options.

Second, launch campus-wide awareness campaigns that divert students away from relatives and social media to credible sources (doctors, university health center, verified online modules). Use multilingual materials, peer-led workshops, and short videos to reach large undergraduates and international subgroups.

Third, establish routine, minimum-cost screening programs for high-risk students with reporting comorbidities or family history, combined with immediate referral pathways. This would help to prove the true prevalence and reduce the over-reliance on self-diagnosis.

Fourth, create a supportive campus environment by improving cafeteria menus, expanding free fitness facilities, and offering dietary counseling through the university health center. Finally, implement ongoing monitoring and evaluation of all initiatives using pre–post surveys and focus groups to ensure sustained behavior change.

Fifth, address practical barriers to healthy practices by conducting formative research (focus groups, surveys) to identify specific obstacles students face in adopting dietary changes and physical activity. Based on these findings, implement environmental modifications such as subsidized healthy meal options in campus cafeterias, extended gym hours, free nutrition counseling, and peer-support groups for students who manage NAFLD or are at high risk. Take advantage of the finding that 86% students reported improvement after lifestyle changes by creating a peer ambassador program where students who successfully modified behaviors can share experiences and strategies with others.

These measures, if implemented, could transform IUT into a model for the prevention of NAFLD among young adults in resource-limited settings.

## CRediT authorship contribution statement

**Salma Khatun:** Writing – review & editing, Writing – original draft, Visualization, Software, Resources, Project administration, Methodology, Investigation, Funding acquisition, Formal analysis, Data curation, Conceptualization. **Iffat Tasnim Haque:** Writing – review & editing, Validation, Supervision.

## Declaration of Generative AI and AI-assisted Technologies in the Manuscript Preparation Process

During the preparation of this work, the authors used Claude (Anthropic) for language editing and improving the readability and structure of the manuscript. The authors reviewed and edited the output as needed and take full responsibility for the content of the published article.

## Funding

This research did not receive any specific grant from funding agencies in the public, commercial, or not-for-profit sectors.

## Declaration of competing interest

The authors declare that they have no known competing financial interests or personal relationships that could have appeared to influence the work reported in this paper.

## Data Availability

The anonymized dataset generated and analyzed during the current study is not publicly available in order to protect participant confidentiality, but is available from the corresponding author upon reasonable request.

## Appendix A Supplementary data

MMC S1 Supplementary File S1: Study questionnaire used to assess NAFLD knowledge and practices among university students. MMC S1

MMC S2 Supplementary File S2: Participant informed consent form used in the study. MMC S2

## Footnotes

1 Note: Percentages may not sum to 100 due to rounding. SD = Standard Deviation.

2 Note: Percentages calculated based on the 261 participants who had heard about fatty liver.

3 Note: Participants could select multiple conditions. Percentages based on 278 participants providing at least one valid answer to this question (65 missing).

4 Note: Percentages are calculated within each category row. Several subgroups contain small cell counts; chi-square results among diagnosed patients should be regarded as exploratory.
