# Supplementary material for: Knowledge and practices related to non-alcoholic fatty liver disease among university students in Bangladesh
Source: Dialogues Health. 2026 Aug 4;9:100334. doi: 10.1016/j.dialog.2026.100334 (PMC13476630; doi:10.1016/j.dialog.2026.100334)
Supplement: Supplementary file 1 [file mmc1.pdf]

Public Awareness of Non-alcoholic fatty liver

Thank you very much for your time and participation in this survey.

1.Age

□ <20yrs □ 20-30yrs □ 31-40yrs □ 41-50yrs

2.Gender

□ Male □ Female

3.Nationality

□Bangladeshi □North American □European

□Middle east □Asian □African

4.Educational Qualification

□ Under Graduate □ Post Graduate □ Masters □ PhD

5.Have you ever heard about fatty liver?

□ Yes □ No

6. If yes, from where have you heard about fatty liver?

□ Doctor □ Relatives □ Social Media □ Google

7.Have you ever heard about cirrhosis (shrinkage of liver)?

□ Yes □ No

8.If yes, which of the following do you think can have advance to cirrhosis ? (one or more answers)

□ Alcohol consumption □ Fatty liver □ Hepatitis C infection □ Hepatitis B infection

If any other option please specify

…………………………………………………………………………………………………

9.Which of these conditions do you think can cause fatty liver? (one or more answers)

□ Obesity □ Diabetes □ Excess alcohol intake □ High cholesterol

10.Do you think fatty liver is hereditary?

□ Yes □ No □Don't know

11.Is there any treatment available for fatty liver?

□ Yes □ No □ Don't know

12.Do you think fatty liver is preventable?

□ Yes □ No □ Don't know

13.Do you think that fatty liver can occur in non-alcoholic?

□ Yes □ No □ Don't know

14.Do you think that fatty liver can be cured in it's early stage?

□ Yes □ No □ Don't know

15.How do you think doctors diagnose fatty liver?

□ Sonogram of liver □ Blood tests □ Body weight/obesity □ Don't know

16.Do you think fatty liver can cause a serious health problem?

□ Yes □ No □ Don't know

17.Have you ever been diagnosed with NAFLD?

□ Yes □ No

18.If yes, do you follow any specific dietary restriction related to NAFLD(Non-Alcoholic Fatty Liver Disease)?

□ Yes □ No □ Don't know

19.Do you have any other existing medical conditions (e.g. diabetes, hypertension) that are related to NAFLD?

□ Yes □ No

20.Are you taking any medications or supplements for liver health of NAFLD management?

□ Yes □ No

21.Did you change your lifestyle to reduce the risk or manage NAFLD?

□ Yes □ No

22. If yes, have you noticed any improvement after changing your lifestyle to reduce or manage NAFLD?

□ Yes □ No
