# Supplementary material for: Knowledge and practices related to non-alcoholic fatty liver disease among university students in Bangladesh
Source: Dialogues Health. 2026 Aug 4;9:100334. doi: 10.1016/j.dialog.2026.100334 (PMC13476630; doi:10.1016/j.dialog.2026.100334)
Supplement: Supplementary file 2 [file mmc2.pdf]

Informed Consent Form

Knowledge and Practices Related to Non-Alcoholic Fatty Liver Disease (NAFLD) Among University Students

Institution: Islamic University of Technology (IUT), Gazipur, Bangladesh

Investigator / contact: Dr Salma Khatun, Medical Department (salmasushanta@gmail.com)

Ethics approval: Ethical Review Board (ERB), IUT

You are invited to take part in a study on knowledge, attitudes, and practices related to NAFLD among IUT students. If you agree, you will complete a short, self-administered questionnaire (about 5 to 6 minutes).

Participation is entirely voluntary. You may skip any question or withdraw at any time before submitting, without giving a reason and without any effect on your studies. There are no known risks and no invasive procedures.

The questionnaire is anonymous. No identifying information is collected; responses are kept confidential, stored securely, used only for this research, and reported only in aggregate.

I have read and understood the information above, have had my questions answered, and voluntarily agree to take part in this study.

Participant signature: ______________________________ **Date:** ________________

Researcher signature: ______________________________ **Date:** ________________
